# Investigating uncharacterised genes in *Saccharomyces cerevisiae* using robot scientists

**DOI:** 10.1038/s41598-026-46236-z

**Published:** 2026-03-31

**Authors:** Erik Y. Bjurström, Alexander H. Gower, Praphapan Lasin, Otto I. Savolainen, Ievgeniia A. Tiukova, Ross D. King

**Affiliations:** 1 Department of Life Sciences, Chalmers University of Technology, Göteborg, Sweden; 2 Department of Computer Science and Engineering, Chalmers University of Technology, Göteborg, Sweden; 3 Department of Clinical Nutrition, University of Eastern Finland, Kuopio, Finland; 4 Division of Industrial Biotechnology, KTH Royal University of Technology, Stockholm, Sweden; 5 Department of Chemical Engineering and Biotechnology, University of Cambridge, Cambridge, UK; 6 Alan Turing Institute, London, UK

**Keywords:** Biotechnology, Computational biology and bioinformatics, Genetics, Molecular biology

## Abstract

Despite extensive research on *Saccharomyces cerevisiae* functional genomics, approximately 880 out of ~6,000 open reading frames (ORFs) remain uncharacterised. In this study we propose a method for characterising genes with limited prior functional knowledge using an automated laboratory platform, in conjunction with several hypothesis instantiation methods. We demonstrate this method by investigating *YGR067C*, an uncharacterised ORF hypothesised to regulate respiration during the diauxic shift. Predictions of the first-order effects of deletion were obtained by curating a list of pathways relevant to the hypothesis. Higher-order effects were predicted using simulation models based on the GEM Yeast9. The predictions were tested using empirical data from biological experiments performed in the Robot Scientist Eve, which generated OD_560_, transcriptomics, and metabolomics data. We observed that *YGR067C* deletion led to downregulation of transcripts in some ethanol consuming respiratory pathways during the glucose phase During the ethanol phase we observed that NAD+, NADP+ and NADH accumulated, and several amino acid biosynthesis pathways were enriched for the *ygr067c∆* strain, suggesting longer term consequences of *YGR067C* mediated regulation. Based on these observations we propose that the role of *YGR067C* during the diauxic shift is to regulate genes related to ethanol consumption and respiration in the glucose phase.

**Supplementary Information:**

The online version contains supplementary material available at 10.1038/s41598-026-46236-z.

## Introduction

*Saccharomyces cerevisiae* is the most studied eukaryotic model organism. Despite this, there are many genes whose biological function is not understood, and a complete understanding of the yeast cell is still far from being achieved^1^. The rate at which we progress toward this goal is limited by human capacity for experimentation and experiment design. To increase the effectiveness of systems biology, we can use computational techniques to select or refine hypotheses in a way to maximise information gain, while minimising the time and economic costs, and we can use laboratory automation to increase the quality of empirical data. These are both important aspects for the wider goal of closed-loop automation of functional genomics. In this study we test and compare computational techniques for refining abstract hypotheses, applying them to investigate the biological role of an uncharacterised open reading frame (ORF) in *S. cerevisiae*.

When investigating an unknown gene with a hypothesised regulatory function, the first approach would be to identify genes and pathways expected to be directly affected by its deletion. However, we anticipate onward effects across the whole organism from the regulation by the gene of interest, instead of just the immediately affected pathways, metabolites, and genes. To predict these higher-order effects of the knockout we need to perform simulations. And to perform simulations we need a computational model of yeast. There are several computational models available^2^, and which to use depends on several factors, including that we require that inputs and outputs correspond to experimental variables that we can control or measure. We use a flux balance analysis (FBA) model and a first-order logic model, LGEM^+^^3^. The predictions and simulations are then compared against newly generated empirical data to verify the hypothesis.

**Fig. 1:**
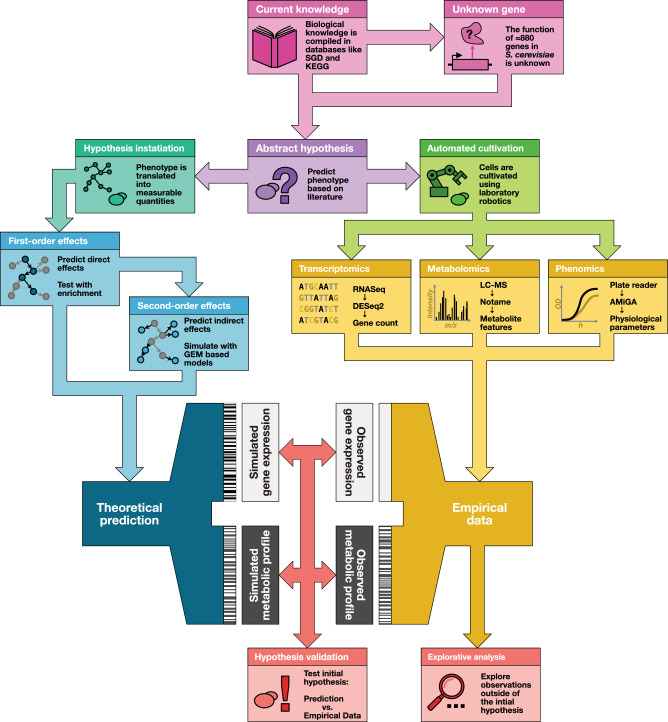
Instantiation of an abstract hypothesis. To be able to test a hypothesis, we need to transform it into data that is comparable with empirical data. This instantiation is usually accomplished using a mathematical model; the choice of model, and of the parameters, will change the predictions. To obtain an interface between prediction and measurement where a statistical test can be used, it is most often necessary to transform both the raw model outputs and the raw measurement data. These additional levels of processing risk weakening the signal and amplifying the noise, leading to increased uncertainty in the test, but also enable the opportunity to compare predictions from different models across different measurements.

Obtaining high quality empirical data to test the predictions is complicated by the nature of the hypothesis. Some genes are only active during metabolic transitions, such as the diauxic shift^4,5^. This necessitates working in dynamical systems, i.e. batch growth, as it would be impossible to observe this effect in stabilised systems such as chemostats^6^. In batch growth, minor fluctuations in initial conditions and sampling time can lead to significant deviations in the empirical data, which complicates statistical inference and affects the reproducibility of the experiment. To address this problem, robot scientists (sometimes “self-driving labs”) have been developed to automate biological experiments to generate highly reproducible scientific results^7–10^. This study used the robot scientist Eve^11^ to cultivate *S. cerevisiae* and generate multiomics data from a diauxic shift experiment. See Fig. 1 for a schematic of the workflow in this study.

We investigated the role of *YGR067C*, an uncharacterised ORF whose protein product functionality is unknown. What is known is that the gene product contains a zinc finger motif, similar to that of Adr1p, which is a respiratory transcription factor active during the diauxic shift in *S. cerevisiae*^12,13^*.* The diauxic shift is a metabolic network rewiring event in *S. cerevisiae*, in which the cell goes from fermentative consumption of glucose and production of ethanol, to respiratory consumption of ethanol once the glucose is depleted^14^. During the first growth phase, known as the glucose phase, respiratory genes are repressed by the transcription factor Mig1p^15^. As the glucose levels decrease, Mig1p is phosphorylated by Snf1p, relieving the respiratory genes of its repression^15^. Adr1p, an activator of respiratory genes such as ADH2, is also activated by Snf1p as the glucose levels decrease^13^. Furthermore, Espinosa et al. observed truncations of YGR067Cp in S. cerevisiae strains evolved to assimilate methanol through adaptive laboratory evolution, and suggested that genes and metabolic fluxes that were favourable for growth on methanol were repressed in the presence of a functional *YGR067C*^16^. Thus, based on previous studies^7^ and the structural similarity between YGR067Cp and Adr1p, we hypothesised that YGR067Cp acts as a transcription factor that regulates respiratory genes during the diauxic shift.

In summary, the aim of this study was to develop a method to investigate uncharacterised genes with little prior knowledge. We demonstrate this by investigating *YGR067C*, a poorly understood gene in *S. cerevisiae* thought to be involved in the diauxic shift. We employed several hypothesis instantiation methods to generate predictions, which were then evaluated using empirical data obtained from experiments performed using the Robot Scientist EVE (an automated laboratory platform). The performance of the model-driven approaches showed that there is room for improvement in the techniques and models. The results from this study suggest that *YGR067C* has a role in regulating respiratory genes during the diauxic shift.

## Results

We hypothesised that the absence of *YGR067C* would disrupt respiratory pathways during the diauxic shift in *S. cerevisiae.* To generate the empirical data necessary for testing our hypothesis, a *ygr067c∆* strain with a reference strain BY4741 was cultivated in the automated laboratory platform Eve. The cells were grown in minimal media with low glucose content (1.25 g/L) to ensure diauxic growth, see Methods section for detailed composition. Glucose phase and ethanol phase samples were taken after 12- and 24-hours post-inoculation, respectively. The sampling times were determined from a previous study^7^. Transcriptomic samples were obtained using RNAseq (transcriptomics) and metabolomic samples through liquid chromatography-mass spectrometry (LCMS), see Methods section for further details.

### Pathway set prediction

The first order effects of the hypothesised disruption should be observable in the transcription of genes and the metabolism in pathways associated with respiration. A first order prediction was generated by curating a list of genes and metabolites recorded in the Kyoto Encyclopedia of Genes and Genomes (KEGG) database^17^ which were associated with respiration, see Methods section. Univariate statistical tests were performed: DESeq2^18^ for transcriptomic data and notame^19^ for metabolomic. The hypothesis was then tested on the transcriptomic data using consensus gene set enrichment, which included Fisher’s exact test, Boschloo’s test^20^, and Fast Gene Set Enrichment Analysis (FGSEA)^21,22^. Individual genes and metabolites within the KEGG pathways were also examined.

During the glucose phase, some respiratory pathways—namely the TCA cycle, oxidative phosphorylation, and the glyoxylate shunt—were over-represented among differentially expressed genes (FDR < 0.05, Fisher’s and Boschloo’s test), see Table 1. FGSEA analysis indicated that these pathways were collectively downregulated, see Table 1. This suggests that transcription was induced by the protein product of *YGR067C* during glucose rich environments. However, the transcripts of genes unique in the fermentative glycolysis pathway (alcohol dehydrogenases and the pyruvate decarboxylase complex) and genes in the respiratory glycolysis pathway (mitochondrial pyruvate importers and the pyruvate dehydrogenase complex) were not over-represented, see Table 1. Genes in the gluconeogenesis pathway were statistically over-represented during the glucose phase using methods with significance cutoffs (Fisher’s and Boschloo’s tests) but not when using FGSEA which considers all genes within a set, see Table 1. Within gluconeogenesis, the differentially expressed genes (DEGs) *PYC1* (carboxylase responsible for conversion of pyruvate to oxaloacetate) and *FBP1* (phosphatase responsible for conversion between fructose-1,6-bisphosphate and fructose-6-phosphate) were significantly downregulated (FDR < 0.05, Wald’s test with BH correction). The remaining genes in this pathway showed positive log_2_ fold-change (log_2_FC) but did not reach significance (FDR < 0.05, Wald’s test with BH correction).

**Table 1:** Consensus gene set enrichment of respiratory pathways using data from differential gene expression analysis. Columns describe the statistical test while rows describe phase and pathway. Fisher’s test and Boschloo’s test are two-tailed hypothesis tests where genes with a FDR < 0.05 were considered significantly differentially expressed. Only distinct regulation was considered for FGSEA and the directions were split into two separate metrics (up- and downregulation). Enrichment analyses with p < 0.05 were considered statistically significant and are marked with bold.

		Fisher’s test	Boschloo’s test	FGSEA up	FGSEA down
Glucose phase	TCA cycle	**7.11E-09**	**5.22E-09**	NA	**2.22E-03**
	Oxidative phosphorylation	**4.89E-05**	**4.27E-09**	NA	**1.73E-02**
	Glyoxylate shunt	**3.45E-06**	**2.38E-06**	NA	**2.22E-03**
	Glycolysis (fermentation)	1	1	NA	7.60E-01
	Glycolysis (respiration)	1	1	NA	1.06E-01
	Gluconeogenesis	**4.48E-02**	**3.94E-02**	NA	1.35E-01
Ethanol phase	TCA cycle	1	1	NA	9.03E-02
	Oxidative phosphorylation	1	9.33E-01	NA	2.02E-01
	Glyoxylate shunt	1	8.76E-01	8.72E-01	NA
	Glycolysis (fermentation)	1	1	2.62E-01	NA
	Glycolysis (respiration)	1	1	NA	9.03E-02
	Gluconeogenesis	1	1	8.72E-01	NA

The transcription of the genes within the predicted pathways were generally downregulated in the deletion mutant during the glucose phase. Only *VMA9* and *VMA10*, both subunits of the H^+^-ATPase complex, were both upregulated and part of the KEGG pathway oxidative phosphorylation .

Of the 85 metabolites included in first order prediction set, only 14 could be identified in the empirical data using peak identification software, and of those only 9 passed the quality control during the pre-processing step, see Supplementary Table 1. This was perhaps not surprising as many of the predicted metabolites are short chain carboxylic acids, which are known to be difficult to detect in conventional LCMS methods due to bad reverse phase retention and inefficient electrospray ionisation^23^. During the glucose phase, the abundance of phosphoenol pyruvate (p = 0.077, log_2_FC(mutant/reference) = -0.0805, linear model) and glutamic acid (p =0.020, log_2_FC(mutant/reference) = 0.87, linear model) were significantly different in the mutant compared to the reference. During the ethanol phase, increased accumulation of nicotinamide adenosine dinucleotides could be observed in the deletion mutant compared to the reference strain: NAD+ (p = 0.024, log_2_FC(mutant/reference) = 2.82, linear model), NADH (p= 1.9 x 10^-4, log_2_FC(mutant/reference) = 2.82, linear model). However, due to the simultaneous accumulation of both NAD+ and NADH in the deletion mutant during the ethanol phase, the NADH/NAD+ ratio did not shift compared to the reference strain (reference strain: 1.00, deletion strain: 1.00). . Furthermore, increased accumulation of glutamic acid, which is essential for the anabolism, could be observed (p = 0.008, log_2_FC(mutant/reference) = 1.73, linear model) in the ethanol phase.

### Results of the FBA simulation method

Using a FBA model with growth as the objective function, we simulated metabolite presence and gene expression for all metabolites and genes in the Yeast9 model, using a pathway perturbation method to instantiate our hypothesis on *YGR067C* function. As FBA does not simulate metabolite accumulation, metabolite presence means that the compound is predicted to be involved in one or more active reactions. Because of the difference in simulation methods, predictions were made for roughly twice as many metabolites as in the LGEM^+^ method, and for roughly three times as many genes, meaning the pathway coverage is greater than either the pathway set prediction method or the LGEM^+^ method, see Table 2.

**Table 2:** Results of simulation methods († - no support for LGEM^+^ metabolomics simulations as there was not overlap between detected metabolites and predicted).

Simulation method	LGEM^+^		FBA	
Phase	Glucose	Ethanol	Glucose	Ethanol
# Pred. diff expr. genes	37	39	564	554
Direction prediction acc. (genes)	2%	2%	23%	19%
# Pred. diff abund.of metabolites	11	18	444	423
Direction prediction acc. (metabolites)^†^	–	–	22%	24%
Predicted affected pathways (in ygr067c∆)	↑ – ↓ –	↑ pentose phosphate pathway ↓ pyruvate decarboxylation to acetyl CoA	↑ – ↓ glucose fermentation, pyruvate fermentation, glycolysis, very long chain fatty acid biosynthesis	↑ glyoxylate cycle ↓ amino acid and nucleotide biosynthesis

During the glucose phase we predicted that overall, 564 genes would be differentially expressed to some degree in the mutant strain; in the ethanol phase this figure was 554. The pathways predicted to be down-regulated the most during the glucose phase in the mutant were glucose fermentation, pyruvate fermentation, glycolysis, and very long chain fatty acid biosynthesis. In the ethanol phase the pathways predicted by the FBA to be most down-regulated in the mutant were various amino acid and nucleotide biosynthesis pathways, and chorismate metabolism; the pathway predicted most up-regulated was the glyoxylate cycle. In contrast to LGEM^+^, the FBA model predicted that 32 and 28 of the detectable metabolites in the glucose and ethanol phases respectively were differentially expressed in the mutant; the model predicted the direction of these differences with an accuracy of 22%.

Full tables for the predictions with evaluation against transcriptomics and metabolomics data are provided in the supplementary information, see Supplementary Table 1.

### Results of the LGEM^+^ simulation method

LGEM^+^ expresses the graph structure of metabolic networks in mathematical logic, then uses an automated theorem prover to simulate (through logical deduction) activated reactions, metabolites, and genes^3^. Compounds and genes that appear in the LGEM^+^ simulation are those predicted to be present. The simulations are not quantitative, so presence is binary, and metabolite presence in the case of LGEM^+^ is defined in the same way as for FBA simulations. We simulated metabolite presence and gene expression for all metabolites and genes in the Yeast9 model, using the same pathway perturbation as the FBA simulations, see Methods section for details on the LGEM^+^ simulations. Most metabolites and genes were predicted not to be present, or expressed, in either the wild-type strain or the *YGR067C* deletant, see Table 2. Therefore, the predictions were largely that there was no difference between the strains.

The LGEM^+^ simulation extends beyond the localised scope of the pathway set prediction, yielding us a prediction of the second-order effect of the hypothesised consequences of *YGR067C* deletion. During the glucose phase this model predicted 37 genes to be differentially expressed in the *YGR067C* deletant compared to the wild type. Genes involved in glycolysis and pyruvate decarboxylation to acetyl CoA were predicted differentially expressed. Of these, 8 were significantly differentially expressed in the empirical transcriptomic data (FDR < 0.05, Wald test with BH correction), see Supplementary Table 1.

Full tables for the predictions with evaluation against transcriptomics and metabolomics data are provided in the supplementary information, see Supplementary Table 1.

### Results outside the initial hypothesis

Our initial hypothesis was only concerned with a relatively small subset of the recorded data, namely genes and metabolites related to respiration. However, RNAseq and untargeted LCMS attempts to capture the entire transcriptome and metabolome, respectively. Here we present observations regarding the transcriptome and metabolome which was outside of our initial hypothesis. Additionally, since no physiological prediction was made prior to the experiments, this section also covers growth parameters.

**Fig. 2:**
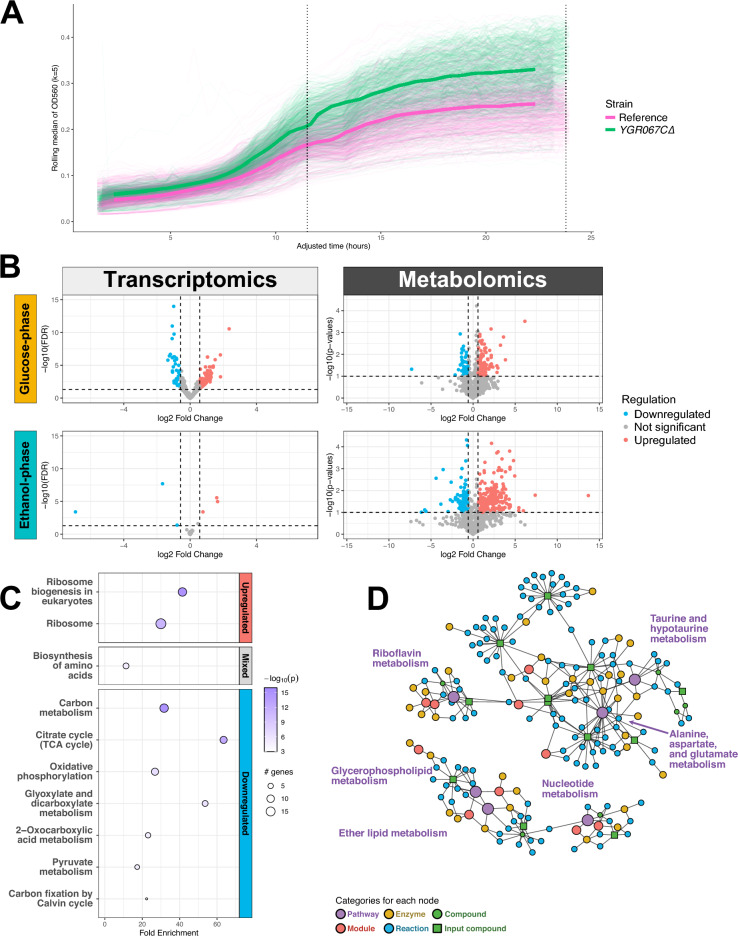
Results outside the initial hypothesis. **A**. Averaged growth curves of the ygr067c∆ strain (pink) and the reference strain (green) with the 11.5-hour and 24-hour sampling points as a dotted, vertical lines. OD_560_ in the y-axis and time in hours in the x-axis. The bold lines represent a rolling median applied over the measured OD_560_. The thinner lines represent the actual OD_560_ measurements from each replicate. **B**. Volcano plots representing differential expression of transcriptomics (left column) and metabolomics (right column) during the glucose phase (top row) and the ethanol phase (bottom row). **C**. Dotplot of pathway enrichment analysis using sub-active networks of the glucose-phase. The top section represents upregulated pathways, middle section mixed regulation, and bottom downregulated pathways. **D**. Diffusion based topological enrichment using FELLA with significantly enriched pathways in red (p < 0.05, FELLA).

The tested EUROSCARF *YGR067C::kanMX4* strain grew faster on average and reached a higher OD_560_ at 24 hours compared to the BY4741 reference strain, see Fig. 2A. Growth curves were obtained by measuring OD_560_ every 20 minutes with OMEGA Polarstar. The growth curves were then used to estimate physiological parameters (maximum biomass specific growth rate during exponential growth, µ, and carrying capacity, OD_560, max_). A permutation test, n = 10,000, was then performed to compare the growth parameters, see Methods section for detailed explanation. For the reference strain, µ was 0.402 h^-1^ and 0.450 h^-1^ for the *ygr067c∆* strain. The ∆µ was therefore 0.048 h-1 and the permutation test yielded p= 0.0001. The OD_560_ at 24 hours post-inoculation was 0.265 for the reference strain and 0.338 for the *ygr067c∆* strain. The mean difference in OD_560_ at 24 hours was thus 0.073 and the permutation test yielded p = 0.0001. The expression of genes responsible for mitochondrial catabolism in the TCA cycle were downregulated in the *ygr067c∆* mutant, see the subsection on pathway set prediction. The increased biomass yield and biomass specific growth rate in the *ygr067c∆* mutant could then perhaps be explained by more energy being funnelled into biomass/population growth instead of mitochondrial proteome investment^24^. Notably however, the cultures in this study were sampled before ethanol could be depleted. It is therefore unknown if the biomass yield in the *ygr067c∆* mutant would remain higher than the reference strain post-ethanol depletion. Finally, no genetic complementation, marker-swap or independent reconstruction of *YGR067C* was performed; causal attribution is therefore provisional.

Our data suggest that the activity of *YGR067C*—which is assumed to be a transcription factor—is more pronounced during the fermentative glucose phase, while the metabolic consequences of *YGR067C* mediated regulation can be observed in both glucose- and ethanol phase. The gene deletion affected gene expression during the glucose phase, while the gene expression is relatively unchanged during ethanol phase, see Fig. 2B. On the other hand, the gene deletion seems to have affected metabolite expression during both phases, see Fig. 2B. Furthermore, in the reference strain, we found that *YGR067C* was significantly under expressed during the ethanol phase compared to the glucose phase (FDR = 4.71x10^-33^, log_2_FC(ethanol phase/glucose phase) = 2.05, Wald test withBH correction).

There were some notable differences in the intracellular metabolism that occurred outside the scope of the original hypothesis, see Table 3.

**Table 3:** Statistically different metabolite abundances between the deletion strain and reference strain in each phase (p < 0.1, linear model) . The metabolites were filtered by the list of compounds found in the yeast9 model. log_2_FC is log_2_(mutant/reference).

	Metabolite	KEGG ID	m/z similarity	log_2_FC	p
Glucose phase	(-)-Riboflavin	C00255	0.99	0.60	0.021
	3',5'-Cyclic AMP	C00575	1	2.94	0.042
	Asparagine	C00152	0.98	1.17	0.083
	Glutamic acid	C00025	0.97	0.87	0.020
	L-Carnosine	C00386	0.85	0.43	0.096
	Phosphoenolpyruvic acid	C00074	0.93	-0.08	0.077
Ethanol phase	(-)-Riboflavin	C00255	0.99	0.41	0.007
	Asparagine	C00152	0.98	0.77	0.085
	Aspartic acid	C00049	1	-0.74	0.090
	Cytidine 5’-diphosphocholine	C00307	1	1.54	0.025
	Glutamic acid	C00025	0.97	1.74	0.008
	Guanine	C00242	0.94	-0.43	0.089
	NAD+	C00003	0.81	2.82	0.024
	NADH	C00004	0.89	2.82	0.000
	NADP+	C00006	1	3.96	0.039
	O-Phosphoethanolamine	C00346	1	-3.56	0.001
	Phenylalanine	C00079	0.86	-1.06	0.057
	Taurocholate	C05122	0.99	1.36	0.078
	Tryptophan	C00078	1	1.48	0.056

While accumulation of NAD+ and NADH was part of the original hypothesis, NADP+ accumulation was not. Interestingly, a significant increase of NADP+ accumulation could be observed in the deletion mutant during the ethanol phase, see Table 3. Unlike the NADH/NAD+ ratio—which did not shift in the mutant—the NADP+/NAD+ ratio increased during the ethanol phase for the mutant (reference: 1.00, mutant: 1.40). Increased accumulation of riboflavin, glutamic acid and asparagine was also observed during both phases in the deletion mutant. Amino acid expression was mixed during the ethanol phase—accumulation of glutamic acid, asparagine, and tryptophan were increased in the mutant while aspartate and phenylalanine abundances had decreased.

To investigate whether pathways outside of the main hypothesis were statistically enriched, topological pathway enrichment was performed on the significantly differentially expressed genes (FDR < 0.05, Wald test with BH correction) and metabolites with significant differences in abundance (p < 0.1, linear model), see Supplementary Table 2. Topological pathway enrichment of the gene expression was performed using pathfindR and only on the DEGs found during the glucose phase as the number of DEGs during the ethanol phase was too small, see Supplementary Table 3. The TCA cycle, glyoxylate pathway, and oxidative phosphorylation, were all downregulated in the *ygr067c∆* strain—compared to the reference strain—during the glucose phase, see Fig. 2C. This was consistent with the hypothesis driven analysis. Furthermore, other pathways related to carbon utilisation which were not part of the hypothesis were downregulated, e.g. the carbon metabolism, 2-oxocarboxylic acid metabolism, and pyruvate metabolism, see Fig. 2C. Ribosome related pathways were significantly upregulated in the *ygr067c∆* strain, Ribosome and Ribosome biogenesis in eukaryotes . Finally, the pathway Biosynthesis of amino acids was significantly enriched but did not have a distinct direction of regulation.

The topological pathway enrichment of the metabolome was performed using FELLA^25^ and both glucose- and ethanol phase datasets, see Supplementary Table 3. During the glucose phase, several signalling pathways were significantly enriched due to the upregulation of 3’-5’-Cyclic AMP in the *ygr067c∆* mutant. Alanine, aspartate, and glutamate metabolism and Riboflavin metabolism were enriched in both phases, most likely due to the increased accumulation of glutamate and asparagine for the prior pathway and increased accumulation of riboflavin for the latter. During the ethanol phase, apart from the previously mentioned pathways, the following pathways were enriched: Glycerophospholipid metabolism , Taurine and hypotaurine metabolism , Ether lipid metabolism, and Nucleotide metabolism see Fig. 2D.

## Discussion

Automation of science is necessary to address the challenges in the quest to fully elucidating the biological function of every gene in *S. cerevisiae*, and other organisms. When automating the scientific cycle, it is necessary to use a model to translate abstract hypotheses into predictable and measurable outcomes. In systems biology the use of mathematical models is particularly important, as each local change (e.g. a gene knockout) will have not only first-order effects on the systems that gene directly interacts with, but higher order effects on other systems in the organism, and these effects are impossible to calculate without a mathematical model. The process of choosing, refining, and applying a model involves taking many decisions. Each of these modelling choices can have a large effect on the predictions, and thus on the evaluation of the hypothesis. To fully automate the scientific cycle, the hypothesis instantiation process must be formalised and recorded.

We hypothesised that *YGR067C* induced respiratory pathways during the diauxic shift based on evidence from previous studies. Pathway set prediction (first-order effects) and model-based approaches such as LGEM^+^ and FBA (higher-order effects) transformed this hypothesis into predictions of differential expression of genes and metabolites. Based on the pathway set prediction it was postulated that a downregulation of respiratory genes and decreased abundances of respiratory metabolites would be observed in the *YGR067C* deletion mutant. The LGEM^+^ simulations also predicted downregulation in respiratory pathways. The FBA simulations predicted a wider effect on metabolism, including: disruption to fermentation pathways during the glucose phase; and down-regulation of various amino acid and nucleotide biosynthesis pathways, and up-regulation of the glyoxylate cycle during the ethanol phase.

The success of a model-driven approach to hypothesis instantiation is dependent on the quality of the model used. Each of the three simulation methods resulted in different predictions, and neither of the model-based predictions worked exceptionally well. LGEM^+^ being a discrete model predicted less disruption than the FBA simulations. A significant modelling challenge was how to translate the abstract hypothesis into the simulation. The technique we used in this study to remove subsets of reactions was chosen because these models only contain metabolic genes and reactions. The low predictive accuracy of both models using this random gene removal indicates this technique needs refinement. A hybrid model, that had representations of signalling and gene regulation connected to the metabolic component, could instantiate the hypothesis differently. Since we hypothesise *YGR067C* to be a transcription factor, developing and implementing a hybrid or whole-cell model, such as that proposed in^16^, and instantiating the hypothesis in the gene regulation part of a simulation would be closer to our hypothesis.

Another challenge in the modelling is how to predict metabolite accumulation and transcript levels. Both the LGEM^+^ and FBA models are qualitative in their predictions, and we arrived at a prediction of up- or down-regulation by averaging over repeated simulations. It would be more desirable to have a model that predicted transcript levels and metabolite accumulation quantitatively. This is the subject of active research^26^. One recent approach that might be worth future investigation is to use a deep-learning model to predict accumulation from flux^27^.

The sampling times for glucose phase and ethanol phase were determined in a previous study^7^. Since the external glucose and ethanol concentrations of the cultivations were not measured during the experiments of this study, we cannot fully discard the possibility that the observed effects were caused asynchronous growth states rather than genotypic effects. However, *ADH2* was significantly upregulated in the 24-hour sample compared to the 12-hour sample for both the reference strain (FDR = 1.23x10^-5^, log2FC(ethanol/glucose) = 1.19, Wald test with BH correction) and deletant strain ( FDR = 6.05x10^-22^, log2FC(ethanol/glucose) = 2.69, Wald test with BH correction), see Supplementary Table 2. This is relevant since *ADH2* is responsible for catalysing ethanol into acetaldehyde (the first catabolic step of respiration using ethanol as a carbon source) and its transcript is induced during the post-diauxic shift^28^. Thus, while we cannot conclusively identify the growth phase of the cells at the time of sampling, the upregulation of *ADH2* indicates that the cells were in the process of/had completed the rewiring of the metabolic network towards the post-diauxic shift phase.

The metabolomics coverage was low compared to the gene transcript coverage. The diversity of metabolites (both in chemical structure and in dynamic range of abundance) posits a difficult challenge for the field of metabolomics^29^. While experimental metabolomics has seen significant advancements over the past years, the precision and coverage of its analytes remain behind that of genomics, transcriptomics, and proteomics. Thus, to accommodate the limited metabolite coverage and high instrument noise of metabolomics, we performed model evaluation using several types of omics data sets, i.e. phenomics, transcriptomics, and metabolomics.

**Fig. 3:**
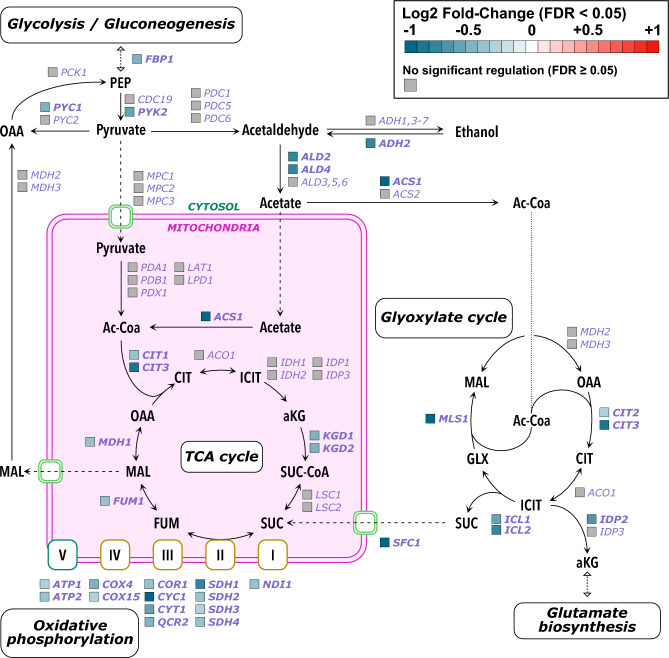
Pathway visualization with transcriptomic regulation of respiratory pathways during the glucose phase. The metabolites are written in black while the genes responsible for the enzymes in the pathway are written in purple. Differentially expressed genes (FDR < 0.05) are written in bold, and the colour of the square represents the log-2 fold change. Genes which were not differentially expressed (FDR ≥ 0.05) are written in plain text and the square is grey. Note that none of the metabolites in this figure were detected in the LCMS.

Pathway set hypothesis testing showed that the TCA cycle, oxidative phosphorylation, glyoxylate cycle were statistically enriched during the glucose phase (p < 0.05; FGSEA, Fisher’s test, and Boschloo’s test) and were furthermore distinctly down regulated (p < 0.05, FGSEA), see Table 1. However, transcription of genes responsible for reactions directing flux from pyruvate to either the TCA cycle or ethanol production were not significantly expressed, see Fig. 3. Instead, genes responsible for irreversible steps in the conversion of ethanol to acetyl-Coa, glyoxylate, TCA cycle, and gluconeogenesis were significantly downregulated, see Fig. 3. Furthermore, many of the downregulated genes were either glucose repressed (e.g. *PYK2*, *ADH2*, *CIT1*, etc)^15^ or induced during consumption of ethanol (*ALD5*, *ALD6*, *PYC1*, etc)^30^. The evidence from this study therefore suggests that it is more likely that *YGR067C* regulates respiratory genes responsible for flux from ethanol rather from glucose, which passes through pyruvate.

There were second-order metabolomic evidence that the glycolytic flux is upregulated in the *ygr067c∆* strain during the glucose phase. Fermentative glycolysis produces organic acids, such as acetic acid, which acidifies the medium and the cytosol^31^. The pH homeostasis is then maintained through V-ATPase-mediated vacuolar acidification^32^. Two subunits of the V-ATPase complex were upregulated in the deletion mutant, which could be an indication towards increased glycolytic flux. Furthermore, metabolomic analysis showed that 3’-5’ cyclic AMP (cAMP) was significantly upregulated in the glucose phase (p < 0.1, linear model). cAMP is a crucial signalling metabolite in the Ras/cAMP-pathway that activates protein kinase A, which in turn regulate many processes related to cell growth, such as the diauxic shift^33^. The Ras/cAMP-pathway is activated by intermediate metabolites in the glycolysis, and the increased cAMP levels could thus be due to the increased glycolytic flux in the deletion mutant^32^.

While respiratory genes were downregulated in the glucose phase, there was no apparent transcriptomic regulation in the ethanol phase. Interestingly however, a significant increase in accumulation of NAD+, NADH, and NADPH was observed in the ethanol phase. Moreover, tryptophan, the precursor molecule for *de novo* NAD synthesis^34^, showed increased accumulation while phenylalanine, which like tryptophan requires chorismite as a precursor^35^, saw decreased accumulation. While the relation between the NADH/NAD+ ratio and fermentation, respiration, and aging have been extensively studied^36^, there does not appear to be much research on increased accumulation of NAD and its derivatives. It is therefore difficult to explain the mechanism behind the NAD+/NADH/NADP+ accumulation based on knowledge from previous studies. One possible explanation would be if the *ygr067c∆* strains had a less developed mitochondria prior to the diauxic shift and was rapidly producing mitochondrial proteins as glucose was depleted. The increased NAD levels could then be explained by the requirement of NADP(H) during amino acid synthesis, and it would also explain the increased levels of glutamate in the deletion mutant.

We found that our hypothesis regarding the role of the uncharacterised ORF *YGR067C* was accurate at a high level, but not specific enough about the predicted effects. The sub-hypothesis, “*YGR067C* induces *ethanol consuming* respiratory pathways *prior to* the diauxic shift” was consistent with the evidence. This lack of specificity in the initial hypothesis has effects on the simulation-based predictions. The accuracy of the simulation-based predictions might have been improved if we had exclusively targeted reactions related to ethanol consumption or had separated the hypothesis into smaller sub-hypotheses, e.g. induction of glucose consuming genes versus induction of ethanol consuming genes.

To conclude, we demonstrate several methods to instantiate hypotheses of uncharacterised genes starting from limited knowledge. The performance of the model-driven approaches showed that the techniques and models require more refinement, which we believe is a worthwhile investment for the future of the field. Finally, based on the results of this study, we suggest that previously uncharacterised ORF *YGR067C* induces ethanol consuming respiratory pathways prior to the diauxic shift.

## Methods and materials

### Pathway set prediction

The hypothesis stated that the transcription of genes and the metabolism in pathways associated with respiration would be disrupted in the absence of *YGR067C*. In the pathway set prediction approach, the hypothesis was instantiated by selecting KEGG pathways that were predicted to be differentially expressed between fermentation and respiration: the citric acid cycle , oxidative phosphorylation, Glyoxylate and dicarboxylate metabolism ), ethanol synthesis from pyruvate , genes exclusively expressed during gluconeogenesis , the pyruvate dehydrogenase complex , and mitochondrial pyruvate carriers (*MPC1, MPC2,* and *MPC3*). The gene and metabolite composition of these pathways sets can be found in Supplementary Table 1. Consensus set enrichment, using Fisher’s test, Boschloo’s test, and Fast Gene Set Enrichment Analysis was then performed FGSEA^20–22^. Fisher’s exact test and Boschloo’s test tends to be overly conservative while FGSEA produces excessive false positives at times^37,38^. Thus, the methods were chosen to provide a balanced the biological interpretation. The consensus set enrichment was then performed on complete KEGG pathways using the predicted gene- and metabolite sets against the empirical transcriptomics and metabolomics data, respectively. The cut-off for the enrichment methods was set to p = 0.05. Individual gene- or metabolite regulation was considered when assessing partial pathway predictions.

### Simulation using LGEM^+^

Metabolic networks can be described in a graph structure which can then be expressed in mathematical logic. Using automated theorem provers we can conduct simulations through logical deduction, and theory repair (hypothesis generation) through abduction^3^. We constructed a first-order logic model of yeast metabolism based on the consensus genome-scale metabolic model Yeast9 (yeastGEM v9.0.2). This model takes as input a given set of available compounds (in this case the minimal growth medium used for the empirical study), and a goal in the form of a subset of metabolites (the production of a set of compounds deemed essential for yeast to grow). Predictions are logical proofs which correspond to activated reactions, metabolites, and genes. As genome-scale models do not inherently model concentration, those compounds and genes that are included in the LGEM^+^ simulation are those predicted to be present. The simulations are not quantitative, so presence is binary.

### Simulation using flux balance analysis

To conduct flux balance analysis simulations, we used the Python library CobraPy (version 0.26.3) with the same version of yeastGEM we used to build the LGEM^+^ model (yeastGEM v9.0.2). The default configuration is for growth in a glucose-rich medium and we used this configuration for the simulations for the glucose phase. For the ethanol phase, we set the bounds for glucose exchange to zero and set the ethanol exchange to be 1.0. We used the default growth objective defined in yeastGEM. To obtain predictions for compounds and genes that are expressed, we took the metabolites and genes associated with each reaction that had a flux greater than a stated threshold (10^-9^ mmol/(g_DW_ * h)) in the found solution. Similarly to LGEM^+^, presence for each simulation is therefore binary.

### Metabolism disruption simulation

*YGR067C* is not present in Yeast9 v9.0.2 which means that simulating the effect of its deletion from the genome is not directly possible with any computational model built upon Yeast9. We also want to avoid using the empirical transcriptomic data to constrain the simulations, as this would introduce a bias in the simulation, we then want to compare our predictions with the empirical data. So, we need another method of introducing the effect of the deletion into the simulation.

In an initial naïve approach, we looked at the compounds and genes in the pathways associated with respiration. For the LGEM^+^ and FBA simulations, we take these same pathways and randomly remove a subset of them before running a growth simulation. This method aims to model the biological effect of a disruption to respiratory pathways, which we hypothesise would be the impact of *YGR067C* deletion. (Note that this method assumes deletion of *YGR067C* would have a negative impact on the respiratory pathways in yeast during the diauxic shift; simulation of a positive regulation after deletion would require a different approach.)

This random deletion is repeated *N*_*sim*_ times, each time removing a subset of reactions of random size between *R*^*-*^_*del*_ and *R*^*+*^_*del*_. Each simulation results in a prediction of the reactions, metabolites, and genes that are activated, see Table 4. We then calculate the difference between the simulation and the non-disrupted pathway.

**Table 4:** Parameters used in the metabolism disruption simulations. * - minimum and maximum number of reactions were found by testing the tolerance of the models to random perturbation, so that the perturbation has a measurable effect on the simulation but does not result in non-growth.

Parameter	Description	Value used	Comment
N_sim_	Number of simulations conducted	500	
R^-^_del_	Minimum number of reactions removed during disruption simulation	5	Found after testing*
R^+^_del_	Maximum number of reactions removed during disruption simulation	12	Found after testing*

Each of these simulations results in a slightly different prediction for the metabolic and transcriptomic activity. Our simulation results are stochastic by nature, the randomness introduced in the size and location of the disruption applied to the model.

The empirical data from growth experiments also have stochasticity. In this case, the randomness arises from many different sources but will vary across cells within the culture. When measuring growth, transcriptomics, and metabolomics, we are measuring the sum of effects of *YGR067C* deletion across all individual cells, smoothing out this stochasticity.

We also sum across our simulations to arrive at data that can be compared to the empirical data, see Fig. 1.

### Strain selection and cultivation conditions

The *S. cerevisiae* wildtype strain BY4741 (Accession number: Y00000) and single-gene deletion strain BY4741 *YGR067C::kanMX4* (Y04697) were taken from the EUROSCARF deletant library^39^. The strains were revived from -80°C glycerol stocks by cultivating them overnight in YPD (2% (w/v) dextrose) media at 30°C, 220 rpm. The strains were then streaked on YPD plates and incubated at 30 ° C for 3 days. Single colonies were then used to inoculate precultures containing YPD (2% (w/v) dextrose) for 15 h at 30°C, 220 rpm. Finally, the main cultivations were performed in Thermo Fisher 384 well MATRIX plates (Thermo 4332), with a working volume of 80 µL YNB medium (10.5 g/L YNB without amino acids, 1.25 g/L glucose, 75 µg/L ampicillin, and 0.625 g/L of L-methionine, L-leucine, L-histidine, and Uracil respectively (Brunnsåker et al., 2023}. Each culture was inoculated with an initial OD_560_ of 0.05, and subsequently incubated at 30°C. Every 20 minutes, the well plate was removed from the incubator, agitated using an orbital shaker, aerated by removing the plate lid, and the OD_560_ was measured using a plate reader (Polarstar). RNAseq and metabolite samples were taken twice, once 12 hours after inoculation, and again after 24 hours. The sampling times were determined from a previous study growing the strains under similar conditions^7^.

### Multiomic extraction and processing

Current RNA extraction protocols and LCMS protocols require biomass concentrations which are not feasible with 80 µL cultures. 96 wells were therefore pooled into one biological replicate for the RNA and metabolite extraction protocols using the liquid handler Bravo. The pooled cell broth meant for RNA extraction was then centrifuged (5,000 rcf, 5 min) and the RNA was immediately extracted using RNeasy kits (QIAGEN). The extracted RNA was stored in 30 µL RNAse free water at -80°C. Total RNA quantity and quality was measured using BioAnalyzer. The library construction and sequencing were performed by Azenta in Leipzig, Germany. Data are deposited at European Nucleotide Archive (PRJEB60302). The raw .fastq files were processed using the nf-core/rnaseq v3.10.1 pipeline^40^, using the *S. cerevisiae* reference genome Ensembl entry R64-1-1, STAR^41^ for fragment alignment and Salmon^42^ for quantification.

The extraction protocol is described in a previous study^7^. Untargeted metabolomics profiling was performed on a Waters Xevo G2-sX qTOF high-resolution mass spectrometers (HRMS) coupled to a Waters Acquity Classic UPLC instrument. Metabolites were separated on an UPLC HSS T3 (1.8 μm, 2.1 × 100 mm, Waters) column with a water-MeOH gradient solvent system containing 0.04% formic acid. The gradient started at 5% MeOH with formic acid (MPB) and ramped to 100% MPB over 6 min and held for 4.50 min at 100% MPB. Column temperature was set to 45 °C and the flow at 0.4 mL/min. Mass spectra were acquired using an electrospray ionization (ESI) source in either positive or negative ionization mode scanning from 40 to 1200 m/z at 5 spectra/second. The capillary voltage was set at 1500 V (ESI negative) and 2000 V (ESI positive), and cone voltage at 40. The source temperature was set at 120°C, desolvation gas temperature at 600°C, desolvation and cone gas flow at 700 and 10 L/min, respectively. Data-dependent MS2 data was collected in both positive and negative ionization by using the following parameters: mass range 40-1200 m/z, MS survey switching threshold 5000, MS survey scanning 0.2 sec, maximum number of precursors 6, scan rate for MS/MS 0.1 sec, collision energy ramp LM CE ramp 6-9 to 60-80 over a mass range of 40-1200 m/z. The raw mass spectra were converted into .mzML files using ProteWizard’s msConvert^43^. Peak picking and initial processing were performed using MSDIAL (v5.4)^44^. Identification was performed using the Riken library of both positive and negative ion mode^44^. The identified peaks were then processed using the Notame^45^ pipeline in R (v. 4.5.0).

### Statistical analysis of empirical data

The phenomic analysis was performed by first compiling the recorded measurements from OMEGA into a .csv file. The .csv file was then used to generate input files compatible for AMiGA^46^. AMiGA then calculates ln(OD_560_) and d/dt ln(OD_560_) at each timepoint t. The carrying capacity OD_560, max_ is obtained by finding the maximum value of ln(OD_560_) during the experiment while the maximum biomass specific growth rate, µ, was obtained by finding the maximum of d/dt ln(OD_560_). Since we did not know the distribution of the biomass specific growth rate and maximum OD_560_, the statistical difference between the reference strain and mutant strain were assessed by performing a permutation test, which is a non-parametric test. The observed test statistic, T_obs_, was calculated by taking the difference in median response value between the strains. The null distribution was then generated by resampling the growth parameter data and recalculating the test statistic n = 10,000 times. The two-sided p-value was then calculated by counting how many times 1 + the absolute value of the sampled permutations exceeded the absolute value of the observed test statistic, divided by n.

The transcriptomics analysis was performed using the DESeq2 software package^18^. Raw expression data, were normalised, fit to a negative binomial distribution, and the log_2_FC of low expression genes was adjusted using the DESeq2-package in R. Hypothesis testing was performed using the Wald test and were corrected for false positives using FDR/Benjamini-Hochberg method with a cut-off of FDR < 0.05. The following contrasts were used for this study: *ygr067c∆* versus reference during glucose phase, *ygr067c∆* versus reference during ethanol phase. The log_2_FC were shrunk using DESeq2’s lfcShrink function with the ‘ashr’ setting^47^.

The metabolomics analysis was performed using the notame package^45^. Univariate significance testing of the identified peaks was performed using linear modelling. The signal intensity was set as the dependent variable while the group (*ygr067c∆* mutant versus reference) was the independent variable. The p-value cut-off was set to p < 0.1, similar to previous studies^48^. This lower level of significance was also chosen partially because of the noise in metabolomics data. From previous experience with this instrument and experimental setup, choosing a stricter significance threshold for this analysis would have reduced coverage. The trade-off for choosing a more permissive significance threshold is that conclusions drawn solely on the metabolomics are weaker, but these data still provide important validation in this study when used alongside the growth data and transcriptomics.

Topological enrichment analysis was performed using active-subnetwork-oriented enrichment analysis through the pathfindR package for the transcriptomic data. For the metabolomics data, a diffusion-based method was performed using FELLA^25^. The protein-protein interaction network used in both topological enrichment analysis was constructed using KEGG graph objects downloaded from KEGG (date). Topological pathway enrichment was performed on the significantly differentially expressed genes (FDR < 0.05, Wald test with BH correction) and metabolites (p < 0.1, linear model).

## Supplementary Information

Supplementary Information 1.

Supplementary Information 2.

Supplementary Information 3.

Supplementary Information 4.

## Data Availability

Data deposition: RNA‐seq data has been submitted in the form of raw reads in the form of .fastq files under the accession number PRJEB60302 at the European Nucleotide Archive (ENA). Metabolomics data has been submitted in the form of derived spectral .mzML files under the accession number MTBLS12663 at the Metabolights.

## Code availability

All code required for reproduction of the analysis and figures in the study can be found on GitHub at https://www.github.com/erikbju/YGR067C_Dshift.
