# Supplementary material for: Investigating uncharacterised genes in Saccharomyces cerevisiae using robot scientists
Source: Sci Rep. 2026 Mar 31;16:10999. doi: 10.1038/s41598-026-46236-z (PMC13043775; doi:10.1038/s41598-026-46236-z)
Supplement: Supplementary file 4 — Supplementary Information 4. [file 41598_2026_46236_MOESM4_ESM.docx]

## Supplementary material

S. Table 1 – Prediction/Simulation of gene deletion vs. empirical data

S. Table 2 – Significantly differentially expressed transcripts and abundant metabolites

S. Table 3 – Topological enrichment (exploratory analysis)
